# Transactions of the American Medical Association

**Published:** 1854-05

**Authors:** 


ART. V. — *Transactions of the American Medical Association for* 1853.

In the brief notice which we have already given to portions of these Transactions we did not intend to forego some subsequent examination of the book as a whole, with more particular reviews of the several monographs it contains.

We have previously noticed the report of the committee on obstetrics, and that of the committee on medical education. But before taking a final leave of them, we have a word of objection to make. The one, we have before spoken of as only a contribution to a particular branch of female disease; the other, as a report, which, though adopted by the association, does not in any sense represent the views of that body, nor, so far as we are informed, of any member of the appointed committee save its chairman. We fear that in both these cases, and certainly in the latter of them, the junior members of the committee, have neglected or avoided a responsibility which it was their duty to assume. These reports should be what they purport to be; they should receive the sanction of the *whole* committee, or, in cases of differences of opinion such as we are aware existed in the committee on medical education, a second report should have been introduced, and its adoption or rejection made a subject of discussion in the association.

The report on medical literature by Dr. N. S. Davis, chairman, embodies a history of the current literature of the day as found in our periodicals, in the transactions of societies, and in the monographs or compends of individual authors. Dr. Davis finds much to commend in our journals, and segregates from them a large number of articles as real contributions to science. In all his instances we believe the praise to have been judiciously bestowed.

Among his criticisms upon the character of our journals, we find the eminently just remark that, while the original and selected departments are marked by good judgment, the department of reviews is almost entirely neglected, consisting in a mere insertion of the title-page of the work under discussion, with a few laudatory remarks attached. In common with nearly all other journals, we must plead guilty, in some measure, to this charge. Excuses enough could be offered were we disposed to present them, but as we have on sundry occasions made some feeble attempts to do justice to the views of authors under review, we prefer to continue these efforts in all cases where time is afforded, or where our own knowledge of a subject fits us for writing upon it.

In his mention of the defects of our literature, Dr. Davis enumerates, first, the insufficient education of many of those who write; second, the disposition to record isolated facts; and third, defective modes of investigation among which he mentions the statistical method.

Of the first of these we, in common with all who are familiar with our periodical literature, are fully conscious. In the second we can see both good and evil. Isolated cases are frequently illustrative. It is not uncommon for the practitioner to date back to some isolated case as one from which he derived much instruction, and upon which he founded some new and successful plan of treatment. If a fact is a fact, if it embodies a new truth, or enforces an old one, if its surroundings are fairly laid before us, we see no harm in its publication, but, on the other hand, consider it valuable just so far as it goes.

The objection to statistical research passes our comprehension. If an isolated fact is worthless, while at the same time statistical collections of facts lead to error, we had better do away with all facts, fall back upon the doctrines of the dogmatists, set up some medical luminary as a prophet, and look upon his works as the Mohammedan does upon the Koran, or the Christian on the Bible; and work by its rules regardless of consequences.

It is an error to suppose that statistics are offered by their collectors as infallible rules. But laws of *probability* may be derived from them of the greatest importance, and the proportions of those probabilities may be stated accurately in figures. The objections to statistics come, so far as we have observed, only from those who are pledged to the identity of typhus and typhoid fevers. It may be interesting to know that the particular statistical records objected to in the report were commenced with the idea of proving their identity. The contrary result obtained is rendered more valuable by this fact.

Leaving here the report of Dr. Davis, we turn to the report on refrigeration produced by upward radiation of heat, as an exciting cause of disease, signed by Drs. Emerson, Hays, and Ruschenberger.

The great merit of this simple and lucid document is in the clearness with which it connects the philosophy and conditions of upward radiation, with the causes which control it. Its application to the causation of disease is not fully explained, but the paper is on the whole a most valuable one, as indicating the phenomena of radiation so clearly as to furnish us every means of warding off its effects.

The changes of temperature effected by radiation, are not regarded as the only, or most prominent, cause of disease. Humidity and malaria are both recognized, and a connection between them and radiation in some degree established.

It is not in our power to do justice by a resume of this paper, but we think our readers will find it one of the most profitable of the various reports included in the volume.

The report of Prof. Gross on malignant diseases has already found mention in our pages. It is not liable to the same objections which pertain to some others. It is a full collection of all attainable facts bearing upon the vexed question of the results of surgical operations in malignant disease. In it Dr. Gross assumes the character of the truly conservative surgeon, and his arguments, enforced and sustained as they are by extensive and reliable statistics, are a sorry comment on that heroic surgery which would extirpate the manifestation of a cachectic condition, while it must leave behind the source and fountain of disease.

The statistics furnished by Prof. Hamilton, of Buffalo, and Warren of Boston, are the most important of the evidences furnished to Dr. Gross by his collaborators. He has very judiciously allowed them to remain in their original form.

The further consideration of these Transactions must be deferred, or perhaps entirely omitted. As a whole, the volume is creditable to the association, and worthy of the profession. H.

